# Apexification in a mandibular first molar with a middle mesial canal

**DOI:** 10.1002/ccr3.1651

**Published:** 2018-06-19

**Authors:** Jesús Alejandro Quiñones Pedraza, Jorge Jaime Flores Treviño, Norma Cruz Fierro, Rosalva González Meléndez, José Elizondo Elizondo, Larissa Argentina Zavala Vargas, Daniel Alberto De La Rosa Moreno

**Affiliations:** ^1^ Department of Endodontics and Department of Advanced Dentistry Autonomous University of Nuevo León Monterrey Nuevo León México; ^2^ Department of Advanced Dentistry Autonomous University of Nuevo León Nuevo León México; ^3^ Private Practice Santiago de Querétaro México

**Keywords:** apexification, clinical outcome, incomplete root development, middle mesial canal, mineral trioxide aggregate

## Abstract

Apexification procedures have been widely used to treat teeth with incomplete root development and pulp necrosis. The middle mesial canal (MMC) is an anatomical variation and in most cases represents a challenge during endodontic treatments. In this article, a favorable outcome is reported after apexification in a molar with MMC.

## 1 INTRODUCTION

Apexification has been defined as “a method to induce a calcified barrier in a root with an open apex or the continued apical development of an incompletely formed root in teeth with necrotic pulps”.^1^

The middle mesial canal (MMC) was described by Vertucci and Williams in 1974.^2^ Since then, its clinical management, incidence, prevalence, and morphology have been studied.^3^, ^4^ Nosrat et al^5^ demonstrated that the localization of this anatomical variation improves with the use of magnification and careful tactile techniques. In contrast, failure in its localization and chemomechanical preparation may lead to persistent disease.^6^

When pulp necrosis occurs in immature teeth, root development may be arrested.^7^, ^8^ Endodontic procedures in these teeth are often challenging due to thin root walls and open apices.^9^ Moreover, the loss of an immature permanent tooth in patients with mixed dentition may cause loss of function, occlusion disorders, and inadequate maxillofacial development.^10^ The purpose of this article was to report an apexification in a mandibular first molar with a MMC.

## 2 CASE REPORT

**Figure 1:**
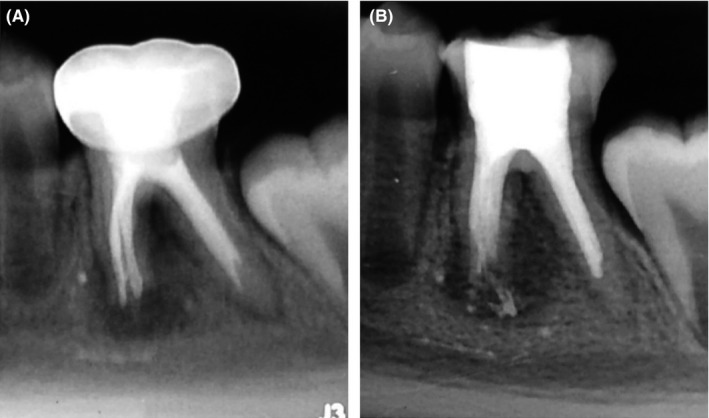
A, Preoperative and (B) postoperative radiographs

A 9‐year‐old girl was referred to the Department of Endodontics of the Autonomous University of Nuevo León for the evaluation of a mandibular first molar. The medical history was noncontributory. The dental history revealed that a root canal treatment had been performed 6 months ago on tooth # 36, and a stainless steel crown was subsequently placed. During the clinical examination, the tooth had a painful response to the percussion and was nonresponsive to the pulp vitality test. Periodontal probing was within the normal limits. A radiographic examination revealed a previous endodontic treatment, periradicular radiolucency, and open apices in the mesial root (Figure 1A). The clinical diagnosis was a previously endodontically treated tooth with symptomatic apical periodontitis, and endodontic retreatment in conjunction with an apexification technique was indicated.

At the first appointment, after administration of local anesthesia with 2% mepivacaine (Scandonest; Septodont), the tooth was isolated with a rubber dam. The stainless steel crown was removed, and access cavity was performed under a dental microscope (Opmi Pico; Carl Zeiss, Oberkochen, Germany). The root canal filling was removed from all the canals with Hedstrom and K3XF files (SybronEndo, Orange, CA), a previously untreated MMC was subsequently located. The working length was established based on radiographs and K‐files (SybronEndo). The instrumentation and irrigation were performed with K3XF files (SybronEndo) and 2.5% sodium hypochlorite (NaOCL). Calcium hydroxide was used as an intracanal medicament.

At the second appointment (after 6 weeks), the patient was asymptomatic. The canals were irrigated with 2.5% NaOCL and 17% EDTA. An MTA (Angelus, Londrina, PR, Brazil) apical barrier of 4 mm was placed only in the mesial canals, and a moist cotton pellet was used to ensure the setting of the material. Calcium hydroxide was applied in the distal canal.

At the third appointment (after 3 days), the presence of a firm barrier of the MTA was assessed using a K‐file. The distal canal and the remainder of the mesial canals were obturated with lateral compaction technique using gutta‐percha and AH Plus sealer (Dentsply DeTrey GmbH, Konstanz, Germany) (Figure 1B). The access cavity was closed temporarily with IRM (Dentsply) at the end of all treatment appointments. Finally, a definitive restoration was indicated.

**Figure 2:**
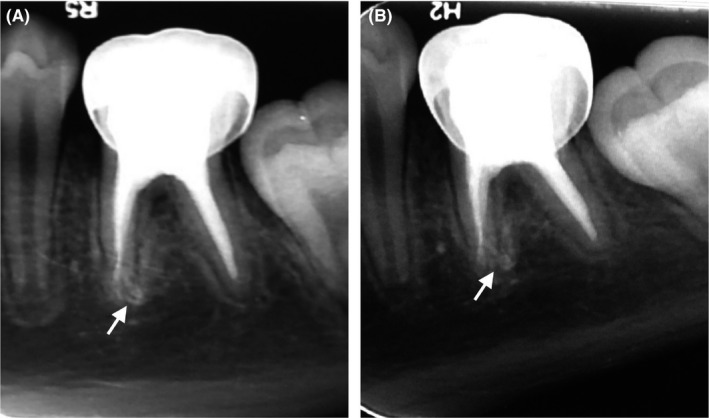
Follow‐up radiographs at (A) 16 and (B) 24 months. Resolution of the periradicular radiolucency and continued root development (arrows) are observed

**Figure 3:**
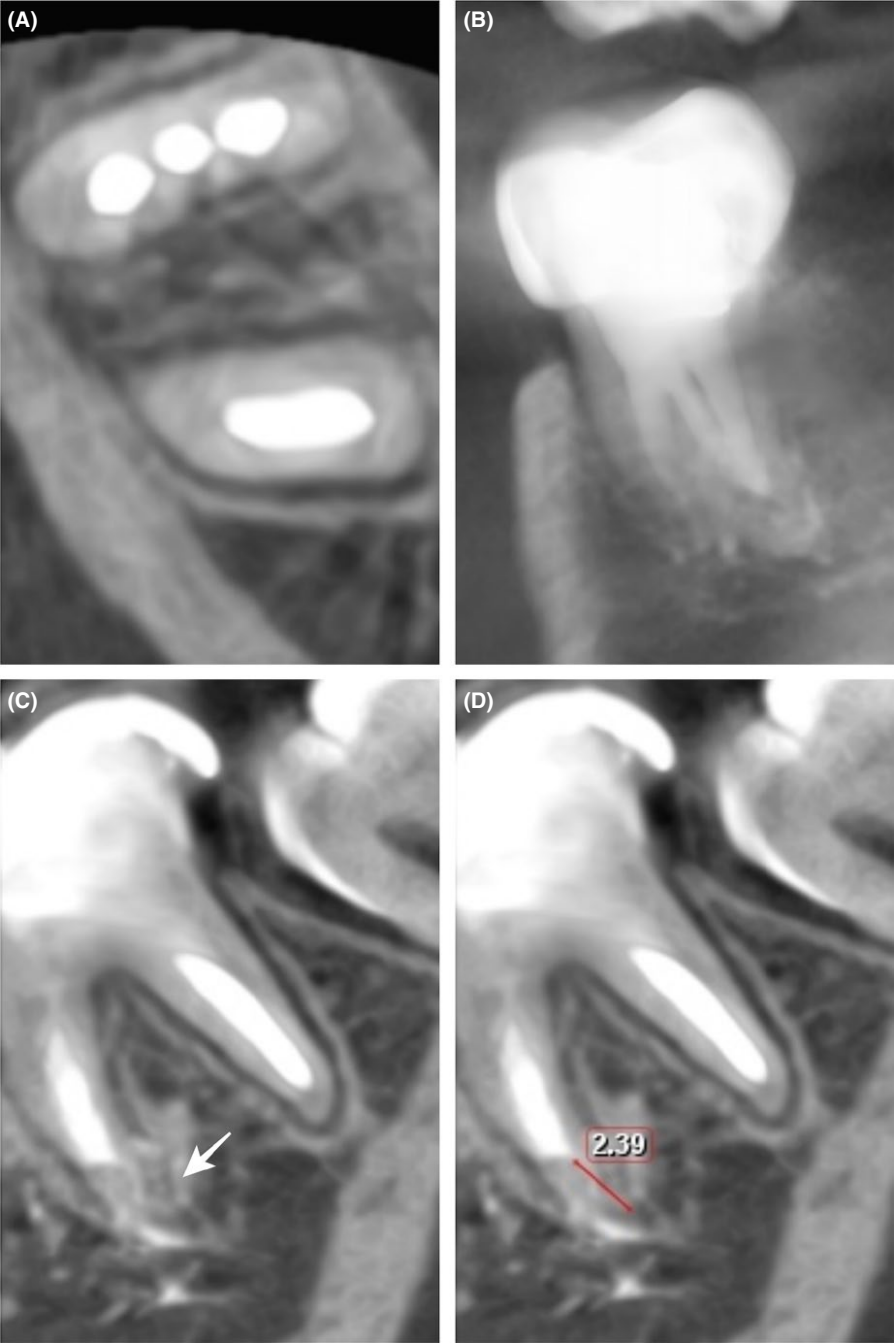
Cone beam computed tomography scan at 24 months. A and B, Obturation of three mesial canals and (C) continued root development (arrow) are observed. D, An approximate measure was obtained from the end of the mineral trioxide aggregate barrier to the end of the root apex

Clinical examination follow‐ups were performed at 16 and 24 months. In both follow‐ups, the patient was asymptomatic. In addition, resolution of periradicular radiolucency and continued root development were observed (Figure 2). At 24 months, a cone beam computed tomography scan (Planmeca Promax 3D; Planmeca, Helsinki, Finland) was performed (Figure 3). An approximate measure of the continued additional root development was obtained using the manufacturer's software (Planmeca Romexis) (Figure 3D).

## 3 DISCUSSION

In this article, a favorable outcome was obtained in terms of disease resolution, tooth retention, and continued root development. Following the fundamental principles of apexification therapy, lesion healing was a crucial step. Likewise, a previously untreated MMC was located during the clinical procedure. The chemomechanical preparation and obturation of this anatomical variation were successfully achieved. After the third treatment appointment, another dentist placed a stainless steel crown. It is important to mention that the second and third treatment appointments were originally indicated at 2 weeks and on the following day, respectively. However, the mother of the patient due to personal reasons delayed such appointments.

Apexification with calcium hydroxide was the most commonly used method for many years. Continued root development of up to approximately 5 mm was reported with this technique.^11^ In our case report, the approximate measure of the additional root development was 2.39 mm. Although calcium hydroxide possesses antimicrobial activity and induces the formation of hard tissue, the main disadvantages of this technique include the multiple visits during a long period of time and the risk of root fracture.

Torabinejad and Chivian^12^ described an apexification procedure using an apical barrier with mineral trioxide aggregate (MTA). MTA provides effective apical sealing in teeth with open apices,^13^ is a biocompatible material with periradicular tissues,^14^ and stimulates the formation of mineralized tissue.^15^ Simon et al^7^ evaluated the clinical outcomes of apexification treatments with MTA. In that study, they demonstrated that this technique is effective in terms of disease resolution and tooth retention. According to previous studies and the clinical outcome of this case report, we suggest that Apexification with MTA can be considered a treatment with favorable prognosis.

Although regenerative endodontic procedure (REP) is also used for teeth with incomplete root development and pulp necrosis, we did not considerate it due to the apex was not open enough in this case. Nowadays, REP and Apexification with MTA are widely used. Paul et al^16^ surveyed endodontists to determine their preference between apexification and regenerative endodontics. Their results indicated that the majority of clinicians preferred apexification treatment when considering the predictability of treatment outcome, whereas regenerative endodontics was preferred when considering the induction of continued root development.

## 4 CONCLUSION

To our knowledge, this is the first case report in the literature about an apexification procedure performed in a mandibular first molar with a MMC. At follow‐up appointments, the patient was asymptomatic. The radiographs and cone beam computed tomography images revealed resolution of the periradicular radiolucency and continued root development.

## CONFLICT OF INTEREST

The authors declare no conflict of interests.

## AUTHORSHIP

JAQP: involved in the management of the patient and drafted the manuscript. JJFT, NCF, RGM, JEE, LAZV, and DADLRM: involved in major revision and in approval of the final manuscript.
